# The impact of video laryngoscopy on the first-pass success rate of prehospital endotracheal intubation in The Netherlands: a retrospective observational study

**DOI:** 10.1007/s00068-022-01962-7

**Published:** 2022-04-01

**Authors:** Iscander Maissan, Esther van Lieshout, Timo de Jong, Mark van Vledder, Robert Jan Houmes, Dennis den Hartog, Robert Jan Stolker

**Affiliations:** 1 Department of Anesthesiology, Erasmus University Medical Center Rotterdam, Rotterdam, The Netherlands; 2 Trauma Research Unit, Department of Surgery, Erasmus MC, University Medical Center Rotterdam, Rotterdam, The Netherlands

**Keywords:** First-pass success rate, Endotracheal intubation, Direct laryngoscopy, Video laryngoscopy, ETI, Prehospital

## Abstract

**Purpose:**

The first-pass success rate for endotracheal intubation (ETI) depends on provider experience and exposure. We hypothesize that video laryngoscopy (VL) improves first-pass and overall ETI success rates in low and intermediate experienced airway providers and prevents from unrecognized oesophageal intubations in prehospital settings.

**Methods:**

In this study 3632 patients were included. In all cases, an ambulance nurse, HEMS nurse, or HEMS physician performed prehospital ETI using direct Laryngoscopy (DL) or VL.

**Results:**

First-pass ETI success rates for ambulance nurses with DL were 45.5% (391/859) and with VL 64.8% (125/193). For HEMS nurses first-pass success rates were 57.6% (34/59) and 77.2% (125/162) respectively. For HEMS physicians these successes were 85.9% (790/920) and 86.9% (1251/1439). The overall success rate for ambulance nurses with DL was 58.4% (502/859) and 77.2% (149/193) with VL. HEMS nurses successes were 72.9% (43/59) and 87.0% (141/162), respectively. HEMS physician successes were 98.7% (908/920) and 99.0% (1425/1439), respectively. The incidence of unrecognized intubations in the oesophagus before HEMS arrival in traumatic circulatory arrest (TCA) was 30.6% with DL and 37.5% with VL. In medical cardiac arrest cases the incidence was 20% with DL and 0% with VL.

**Conclusion:**

First-pass and overall ETI success rates for ambulance and HEMS nurses are better with VL. The used device does not affect success rates of HEMS physicians. VL resulted in less unrecognized oesophageal intubations in medical cardiac arrests. In TCA cases VL resulted in more oesophageal intubations when performed by ambulance nurses before HEMS arrival.

## Introduction

For decades, endotracheal intubation (ETI) has been considered the best way to secure a definitive airway in emergency medicine. This technique can be lifesaving, but its success depends strongly on the first-pass success rate of the provider [1]. This rate in turn depends on provider experience and exposure [1–3], with limited experience associated with a two-fold increase in mortality among patients with traumatic brain injury [4]. A UK study identified an overall ETI success rate of 64% among paramedics, with an unrecognised oesophageal intubation rate of 11% [5].

These and other findings have led to the introduction of alternative airway techniques such as supraglottic airway devices in Scandinavian and British paramedic based emergency medical service (EMS) systems [6, 7]. The new European Resuscitation Council (ERC) guidelines state that only experienced providers should perform advanced airway management during cardiopulmonary resuscitation (CPR) [8]. Less experienced providers should use a supraglottic airway device or perform mask bag ventilation during CPR. In the Netherlands, in contrast to paramedic-based EMS systems in other western countries, specialised nurses staff the ambulances. Data are limited on prehospital intubation success rates in the Netherlands, including comparisons among ambulance nurses, helicopter EMS (HEMS) nurses and HEMS physicians and by direct versus video laryngoscopy [9, 10].

The aim of the current study is to evaluate the first-pass endotracheal intubation rate, overall ETI success rate, and accidental unrecognised oesophageal intubation rate for providers with different experience using direct laryngoscopy compared to video laryngoscopy.

## Methods

This retrospective observational cohort study covers a 6-year period (1 January 2014 to 31 December 2020) using data from the Rotterdam physician-staffed HEMS (P-HEMS) database. The medical ethical commission of the Erasmus Medical Center Rotterdam approved a waiver for this study (MEC-2021-0243).

All consecutive patients were included, and all underwent ETI by direct or video laryngoscopy in the prehospital setting, performed by an ambulance nurse, HEMS nurse, or HEMS physician. Ambulance nurses used the direct or video laryngoscope provided by their ambulance service. HEMS nurses and physicians could choose either at their discretion. Patients with missing data on the number of intubation attempts or tube position were excluded.

The primary outcome measure was the first-pass ETI success rate for each of the three provider groups using direct or video laryngoscopy. Secondary outcome measures were the overall ETI success rates and the rate of unrecognised oesophageal intubation in the three groups using direct or video laryngoscopy. First-pass ETI was defined as a successful placement of a tube in the trachea at the first attempt. An ETI attempt was defined as any attempt in which the laryngoscope blade was passed beyond the lips of the patient. Overall ETI success rate was defined as an effective endotracheal intubation by one provider after one or more attempts. Unrecognised oesophageal intubation was defined as a tube located in the oesophagus by the next airway provider (re)assessing the airway.

Experience with ETI differs among individual Dutch ambulance nurses because of variability in training and prior experience in intensive care, emergency care, or anaesthesia nursing. Before joining an EMS, all nurses undergo an initial 8 month prehospital emergency care training program. If the candidate has no previous airway training or experience, they will be signed up for an ETI training in the operating theatre in an affiliated hospital. These trainings vary from 3 to 8 days depending on logistics and the schedules of the EMS and the hospital. Average prehospital exposure is 3–6 ETIs per ambulance nurse per year [9]. The availability of refresher courses or extra training in airway management is a matter of individual or local priority. Some services invite HEMS physicians or anaesthesiologists to provide additional teaching, training, and tips and tricks, but these additions are not standardised. Dutch EMS vehicles are equipped with a bag-mask-valve, laryngeal masks, Macintosh or video laryngoscope, and tubes for non-drug-assisted ETI.

HEMS nurses have previous ETI experience in their role as an ambulance nurse and perform all their HEMS intubations under supervision of a HEMS physician. Their yearly ETI exposure is around 10–15 cases.

The Rotterdam HEMS crew includes anaesthesiologists (*n* = 10) with considerable experience in airway management (> 3000 ETIs) or trauma surgeons (*n* = 2) with extensive initial HEMS training (> 300 ETIs) in airway management. Yearly exposure to ETI in prehospital settings exceeds 50 per physician. In addition to the standard ambulance equipment, HEMS physicians can administer all advanced airway interventions including prehospital induction of anaesthesia and surgical airway placement. All intubations by ambulance nurses or HEMS nurses in this study that were supervised by a HEMS physician patients recieved proper medication if indicated. To evaluate the position of the tube, HEMS and ground EMS use end-tidal CO_2_ monitoring and clinical evaluation to confirm tracheal placement after ETI. Most intubations in this study by ambulance nurses and HEMS nurses under supervision of a HEMS physician were drug assisted. Only some medical cardiac arrest patients were intubated without medication. All ETI attempts by ambulance nurses before HEMS arrival were non-drug assisted intubations.

The Rotterdam HEMS is dispatched via the ambulance dispatch centre of Rotterdam. Dispatch can be primary, based on caller information about the mechanism of injury or the patient’s clinical condition, or secondary if the ambulance crew requests extra medical support on-scene by a HEMS nurse and physician.

The Netherlands is a densely populated small, well-developed country with a high density of hospitals. The mean distances to a level 1 trauma center is about 30 km by road. The Rotterdam HEMS annualy transports about 3% of their prehospital patients by helicopter. The other patients are transported by road ambulances. If neceserry the HEMS physician will excort the patient after stabilization on scene during the ambulance transportation together with the ambulance nurse.

## Statistical analysis

Statistical analysis was performed using SPSS software (version 25, IBM Corp., Armonk, NY, USA). Normality of continuous data was tested with the Shapiro–Wilk test, and homogeneity of variances was tested using the Levene’s test. Data are reported per group (i.e., intubation by ambulance nurse, HEMS nurse, or HEMS physician). Because all continuous data deviated from normal distribution, we have used the Mann–Whitney U-test for differences among groups and reported the results as medians with quartiles (Qs). Categorical data are reported as numbers with percentages, and differences across groups were tested using the Chi-squared test.

## Results

**Fig. 1:**
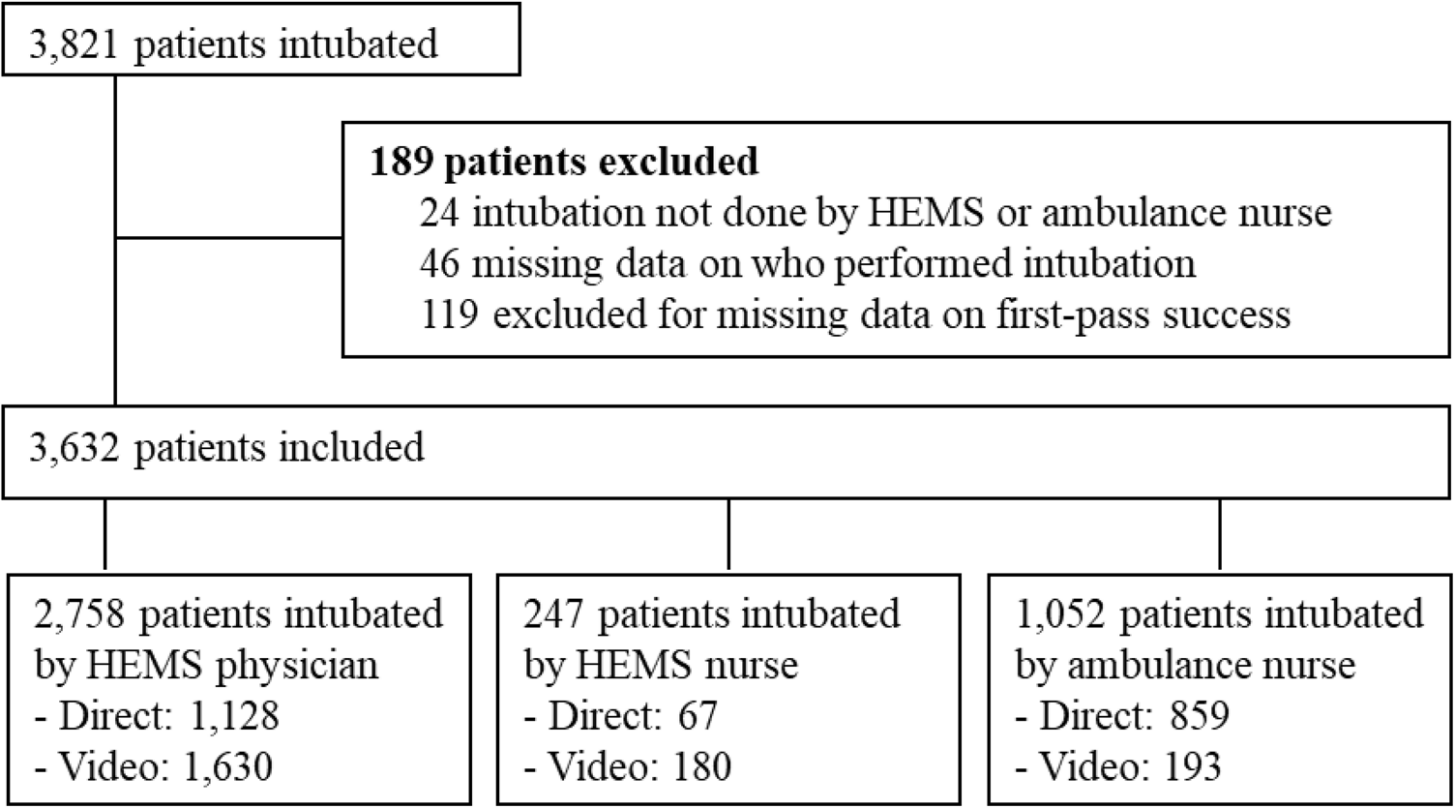
Study flowchart

A total of 3821 patients underwent one or more ETI attempts in the prehospital setting during the study period. Of those, 189 (4.9%) had missing data for first-pass success, identification of the provider performing the intubation, or number of intubation attempts and were therefore excluded from analysis for first-pass success (Fig. 1). Demographic data for the remaining 3632 patients are shown in Table 1. The median age was 51 (Q1–Q3, 30–67) years, with a wide range of 0 to 100 years. The indication for most intubations was a Glasgow Coma Scale < 8 (*n* = 1576; 49.0%) and CPR (*n* = 1345; 41.8%; Table 2). Patient characteristics and indication for ETI were similar between the direct and video laryngoscopy groups.

**Table 1:** Demographics of the study population, grouped by type of provider performing the endotracheal intubation

	All intubations (*n* = 3632)		HEMS physician (*n* = 2758)		HEMS nurse (*n* = 247)		Ambulance nurse (*n* = 1052)	
	*n*		*n*		*n*		*N*	
Male	3632	2414 (66.5)	2758	1853 (67.2)	247	162 (65.6)	1052	694 (66.0)
Median age (years)	3632	51 (30–67)	2758	50 (29–66)	247	53 (33–69)	1052	53 (35–68)
Trauma	3632	1848 (50.9)	2758	1467 (53.2)	247	120 (48.6)	1052	449 (42.7)
Primary dispatch	3591	2597 (72.3)	2726	2002 (73.4)	246	174 (70.7)	1040	712 (68.5)
Cardiopulmonary resuscitation	3632	1345 (37.0)	2758	989 (35.9)	247	75 (30.4)	1052	535 (50.9)
Traumatic cardiac arrest	3632	367 (10.1)	2758	271 (9.8)	247	19 (7.7)	1052	157 (14.9)
Medical cardiac arrest	3632	978 (26.9)	2758	718 (26.0)	247	56 (22.7)	1052	378 (35.9)

Data are shown as *n* (%) or as median (P_25_–P_75_)

*HEMS* Helicopter Emergency Medical Services

**Table 2:** Indications for endotracheal intubation, grouped by type of provider performing the procedure

	All intubations (*n* = 3632)		HEMS physician (*n* = 2758)		HEMS nurse (*n* = 247)		Ambulance nurse (*n* = 1052)	
	*n*		*n*		*n*		*N*	
ETI indication								
Glasgow Coma Scale < 8	3214	1576 (49.0)	2459	1223 (49.8)	224	120 (53.6)	923	351 (38.0)
Cardiopulmonary resuscitation		1345 (41.8)		989 (40.2)		75 (33.5)		535 (58.0)
(Partial) airway obstruction		189 (5.9)		164 (6.7)		20 (8.9)		16 (1.7)
Severe agitation		51 (1.6)		42 (1.7)		6 (2.7)		6 (0.7)
Airway oedema		40 (1.2)		32 (1.3)		2 (0.9)		10 (1.1)
Foreign body		13 (0.4)		9 (0.4)		1 (0.4)		5 (0.5)
Intubation prior to HEMS arrival	1270	246 (19.4)	N.A	N.A	N.A	N.A	1052	246 (23.4)

Data are shown as *n* (%)

*ETI* endotracheal intubation, *HEMS* Helicopter Emergency Medical Services, *N.A.* not applicable

### First-pass ETI success rate

First-pass ETI success rates in the entire group were 49.0% (516/1052) for ambulance nurses 71.9% (159/221) for HEMS nurses and 86.5% (2041/2359) for HEMS physicians.

First-pass ETI success rates with direct laryngoscopy were 45.5% (391/859) and with video laryngoscopy 64.8% (125/193); *p* < 0.0001) for ambulance nurses.

First-pass ETI success rates with direct laryngoscopy were 57.6% (34/59) and with video laryngoscopy 77.2% (125/162); *p* = 0.0042) for HEMS nurses.

First-pass ETI success rates with direct laryngoscopy were 85.9% (790/920) and with video laryngoscopy 86.9% (1251/1439); *p* = 0.488) for HEMS physicians (Table 3).

**Table 3:** First-pass and overall success of ETI in prehospital setting in the Netherlands in patients in whom no previous attempt was made, grouped by type of provider performing the ETI and by device

	HEMS physician		HEMS nurse		Ambulance nurse	
	*n*		*n*		*n*	
All intubations, *n* (%)						
First-pass success	2359	2041 (86.5)	221	159 (71.9)	1052	516 (49.0)
Direct laryngoscopy	920	790 (85.9)	59	34 (57.6)	859	391 (45.5)
Video laryngoscopy	1439	1251 (86.9)	162	125 (77.2)	193	125 (64.8)
Overall success	2359	2333 (98.9)	221	184 (83.3)	1052	651 (61.9)
Direct laryngoscopy	920	908 (98.7)	59	43 (72.9)	859	502 (58 4)
Video laryngoscopy	1439	1425 (99.0)	162	141 (87.0)	193	149 (77.2)

Data are shown as *n* (%)

*ETI* endotracheal intubation, *HEMS* Helicopter Emergency Medical Services

### Overall ETI success rates

Overall ETI success rates in the entire group were 61.9% (651/1052) for ambulance nurses 83.3% (184/221) for HEMS nurses and 98.9% (2333/2359) for HEMS physicians.

With direct laryngoscopy overall intubation success rates were 58.4% (502/859) and with video laryngoscopy 77.2% (149/193); *p* < 0.0001) for ambulance nurses.

Overall intubation success rates with direct laryngoscopy were 72.9% (43/59) and 87,0%(141/162); *p* = 0.0133) with video laryngoscopy for HEMS nurses (Table 3).

Overall intubation success rates with direct laryngoscopy were 98.7% (908/920) for HEMS physicians. With video laryngoscopy the overall success rates for HEMS physicians was 99.0% (1425/1439); *p* = 0.499) (Table 3).

In Table 4 we describe the first-pass and overall success rate per provider and per device used for ETI after prior attempts by another airway provider. After previous attempts of the ambulance nurses the first-pass success of the HEMS nurses and HEMS physicians are lower. The overall success of HEMS nurses in this group is lower as well but the overall success for HEMS physicians was more or less the same as in Table 3 (*p* = 0.19).

**Table 4:** Intubations after previously failed ETI

	HEMS physician		HEMS nurse		Ambulance nurse	
	*n*		*n*		*n*	
*Intubations by HEMS physician or HEMS nurse after failed ETI by ambulance nurse*						
All intubations, *n* (%)						
First-pass success	362	288 (79.6)	26	12 (46.2)	N.A	N.A
Direct laryngoscopy	192	161 (83.9)	8	5 (62.5)	N.A	N.A
Video laryngoscopy	170	127 (74.7)	18	7 (38.9)	N.A	N.A
Overall success	362	355 (98.1)	26	14 (53.8)	N.A	N.A
Direct laryngoscopy	192	190 (99.0)	8	5 (62.5)	N.A	N.A
Video laryngoscopy	170	165 (97.1)	18	9 (50.0)	N.A	N.A
*Intubations by HEMS physician after failed ETI by HEMS nurse*						
All intubations, *n* (%)						
First-pass success	46	30 (65.2)	N.A	N.A	N.A	N.A
Direct laryngoscopy	18	14 (77.8)	N.A	N.A	N.A	N.A
Video laryngoscopy	28	16 (57.1)	N.A	N.A	N.A	N.A
Overall success, all intubations	46	44 (95.7)	N.A	N.A	N.A	N.A
Direct laryngoscopy	18	18 (100.0)	N.A	N.A	N.A	N.A
Video laryngoscopy	28	26 (92.9)	N.A	N.A	N.A	N.A

*ETI* endotracheal intubation, *HEMS* Helicopter Emergency Medical Services, *N.A.* not applicable

When HEMS nurses failed to intubate, the first-pass success of HEMS physicians was significantly (*p* < 0.0001) lower but the overall success was only slightly (*p* = 0.045) lower than in Table 3. HEMS physicians had lower overall success rates with video laryngoscopy 92.9% (26/28) in patients with prior attempts by other airway providers compared with making their own first attempt 99.0% (1425/1439); *p* = 0.0024; Table 4).

### Unrecognised oesophageal intubation

The database also contained information on oesophageal intubation in patients already intubated before HEMS arrival. Ambulance nurses intubated 246 patients before HEMS arrived on scene. In this subgroup of 246 patients, 17.1% (42/246) had an unrecognised oesophageal intubation, requiring intervention by a HEMS physician. In traumatic circulatory arrest cases a substantial number of patients was intubated in the oesophagus with both direct (37.5%) and video (30.6%) laryngoscopy. If direct laryngoscopy was used for intubating patients in medical cardiac arrest, 20% of attempts resulted in unrecognised oesophageal intubations. If video laryngoscopy was used in medical cardiac arrest cases, no unrecognised oesophageal intubations were reported (Table 5).

**Table 5:** Prehospital intubations by ambulance nurse prior to HEMS arrival and percentage of unrecognized esophageal intubations (malposition)

	*n*	Malposition
All intubations (all indications)	246	42 (17.1%)
Traumatic circulatory arrest	65 (26.4%)	21 (32.3%)
Medical CPR	115 (46.7%)	18 (15.7%)
Other	66 (26.8%)	3 (4.5%)
All intubations for traumatic circulatory arrest	65	21 (32.2%)
Direct laryngoscopy	49	15 (30.6%)
Video laryngoscopy	16	6 (37.5%)
All intubations for medical CPR	115	18 (15.7%)
Direct laryngoscopy	90	18 (20.0%)
Video laryngoscopy	25	0 (0.0%)

All intubations by HEMS nurses reported in this study were performed under direct supervision of the HEMS physician, and no oesophageal intubations occurred in this group. One patient was intubated in the oesophagus by a HEMS physician (1/2459; 0.0004%) which was recognized by the anaesthesiologist of the trauma team in the receiving hospital.

## Discussion

The results of this study show a higher first-pass and overall success rate for ETI by HEMS physicians than HEMS nurses and ambulance nurses. HEMS nurses performed better than ambulance nurses in both first-pass and overall success rate. This indicates a beneficial effect of provider experience and exposure in first-pass and overall success rates of ETI in prehospital setting.

Both HEMS nurses and ambulance nurses had a higher first-pass and overall success rate with videolaryngoscopy compared to direct laryngoscopy. For HEMS physicians the type of device used for ETI did not affect their first-pass or overall intubation success rate.

In contrast, with our results, Breeman et al. reported that the use of video laryngoscopy did not improve first-pass success rates and only slightly increased the overall success rate during CPR for medical cardiac arrests in their cohort of Dutch ambulance nurses intubating without supervision of a HEMS physician [10].

Of all 1052 intubations performed by ambulance nurses 246 patients (23.3%) were intubated before HEMS arrival. Despite the use of end tidal CO_2_ measurement 17.5% (43/246) of patients were intubated and ventilated in the oesophagus. (Table 5) Of these cases 73,1% (180/246) were in a circulatory arrest (Traumatic circulatory arrests or medical cardiac arrests) in which ETCO_2_ levels are lower and probably less predictive for correct tube position. In cases of traumatic arrests the video laryngoscope resulted in more unrecognized oesophageal intubations than direct laryngoscopy. Blood and other secretions may have covered the lens making the Mc Graft laryngoscope just an ordinary direct Macintosh laryngoscope. The incidence of oesophageal tubes in traumatic circulatory arrests was 30.6% (15/49) when a direct laryngoscope was used and 37.5% (6/16) after video laryngoscopy. Intubation with a direct laryngoscope in cases of medical cardiac arrest resulted in 20% unrecognized oesophageal intubations. All malpositioned tubes were repositioned after HEMS arrival. One should be cautious in drawing conclusions because the numbers are low in this subgroup but video laryngoscopy seems to lower the frequency of unrecognised oesophageal intubations during CPR for 25 registered cases in medical cardiac arrest.

In Dutch prehospital care, two ambulances are dispatched for medical cardiac arrest, without the primary assistance of HEMS. The Difficult Airway Society and the recently published ERC guidelines advise airway management with supraglottic devices or bag-valve-mask ventilation during CPR by pre hospital personal based on the results of the AIRWAYS-2 trail [8, 11, 12]. This study was performed in the United Kingdom (UK) including nearly 9300 adults in medical cardiac arrest in need of airway management during CPR. No significant difference on patient outcome was found between supraglottic airway devices and endotracheal tubes [11].

These paramedics in the UK, with no prior medical education, have a 30-month initial training programme with a 2 week hospital placement in which they have to perform 20–25 ETI’s under direct supervision. In contrast Dutch ambulance nurses may have more experience in airway management if they have had prior exposure as an anaesthesia nurse. However the colleague’s with prior experience at an intensive care unit or emergency department have less previous experience in advanced airway management and are less skilled than their colleagues from the UK in ETI. From that perspective the Dutch protocols should be adjusted to the new ERC guidelines as well when it comes to airway management by these lower skilled colleagues.

If the Dutch EMS decides for some reason to stick to the current approach; ETI as their first choice during CPR, the training of the providers should be greatly expanded to increase the first-pass and overall success rates. Reduced physiological reserves in critically ill patients contributes dramatically to added risks in non-elective settings, causing profound peri-intubation hypoxia, arrhythmia, cardiac arrest, and death [13]. Repetitive ETI attempts during CPR may take away effort and attention from uninterrupted thoracic compressions and electrical defibrillation [8].

Buis et al. found that a threshold of 50 ETIs with direct laryngoscopy in elective circumstances is needed to reach a success rate of 90% in one or two attempts [14]. Prior exposure of at least 240 intubations with direct laryngoscopy is required to achieve a 90% success rate during CPR [15]. As to the results of our study, using video laryngoscopy instead of direct laryngoscopy may improve the first-pass and overall success rates with limited prior exposure. One can argue if the current exposure of 3–6 intubations a year for ambulance nurses in the Netherlands is enough to prevent from so called skill atrophy. Prehospital use of laryngeal masks during CPR has been studied in the Dutch EMS before and seems viable [6, 7, 11, 16]. The first attempt success of insertion of a laryngeal mask by Dutch ambulance nurses is up to 98% and the incidence of regurgitation and aspiration in the ETI and the LMA group were not significantly different in the AIRWAY-2 trial [7, 11, 16].

Although most airways can be managed with supra glottis airway devices, some need urgent intubation when the LMA doesn’t fit good enough and bag-mask-valve ventilation is ineffective. Concentration of advanced airway management skills in a selected group of prehospital care workers like physician-staffed-HEMS or within the EMS like solo rapid responders or physician assistants with previous experience in anesthesia or excessive training may increase annual ETI provider exposure and may increase ETI success rates in cases a LMA is not suitable. This would match the system on the emergency departments in the Netherlands. The Dutch commission for healthcare audit and inspection stated that an advanced airway provider in the emergency department should be on scene in 15 min and should have previous experience and an exposure of at least 50 (non)elective ETI’s a year to be considered current in airway management in non-elective emergency settings [17]. Providers without such exposure should focus on bag-mask-valve ventilation. If this technique fails a LMA can be inserted to optimize ventilation conditions until an airway expert arrives and can intubate the patient.

Capnography is the gold standard for confirming endotracheal tube position, even in a CPR scenario. After its introduction in anaesthesia the number of unrecognized oesophageal intubations decreased dramatically even in cases where bilateral breath sounds were heard, no borborygmus was heard, fogging of the tube and symmetric thoracic movements were observed [18, 19].

Although capnography was used in all medical CPR cases in this study, 20% of patients that were intubated under direct laryngoscopy prior to HEMS arrival had an unrecognised oesophageal tube position. The HEMS database does not contain information to explain why these tube malposition’s went unrecognised despite the use of capnography. From case debriefings, HEMS nurses and physicians recall that the medical condition of the patient or equipment failure was mentioned as the cause of the absence of end-tidal CO_2_. Because the tube position, was never doubted more training for ambulance nurses is needed in interpreting ETCO_2_ waveforms as well [17]. In all patients intubated by or under supervision of a HEMS physician ETCO_2_ curves were evaluated in the field and reported in the database to prove the correct tube position. If tube position was doubted tubes were taken out and bag-mask-valve ventilation was continued until another intubation attempt resulted in a tracheal tube position confirmed with ETCO_2_ waveforms. In the case of unrecognized oesophageal intubation by a HEMS physician the position was evaluated by auscultation and capnography. Bilateral breath sounds were heard after intubation but were in fact referred sounds from air moving up and down the oesophagus [20]. The first capnography waveforms registered directly after intubation probably were attributable to gastric carbon dioxide. After quick evacuation from the hazardous situation in which the trauma patient and the (H)EMS crew were at the time of intubation, a reassessment showed no capnographic waveform anymore. Tube position was not doubted and alternative causes for the absence were treated (e.g., tension pneumothorax and hypovolaemia).

Some Dutch ambulance nurses with limited experience currently use laryngeal masks as their first-choice device, whereas others adhere to the national protocol of ETI with the provided device. Ambulance nurses with prior training in anaesthesia are more experienced in ETI and one can assume they may provide better care by handling a laryngoscope [14]. One region in the Netherlands has changed the protocol and made laryngeal masks the only option for airway management because of low ETI success rates with direct laryngoscopy. In addition, because video laryngoscopy is often thought to improve intubation success rates among low- and intermediate-skilled airway providers, some services have equipped their ambulances with video laryngoscopes [21]. According to our results, equipping all Dutch ambulances with video laryngoscopes could reduce unrecognised oesophageal tube placements by ambulance nurses during medical CPR. Based on the other results in this study the success rates with both direct and video laryngoscopy by ambulance nurses with the current training programme are too low to continue the practise of ETI in CPR cases by ambulance nurses.

## Limitations

This is a retrospective database study caring the risk of several biases and confounders. Furthermore the population in this study does not represent the entirety of patients intubated in the prehospital setting because HEMS is not dispatched for all CPR scenarios in adult patients. Relevant issues on anatomy (small mouth opening, small mandibles, small thyromental distance) or injury-related challenges (fractures of the jaw teeth or CWK) are not registered in the database. This may introduce a bias of more complex cases in the HEMS database. Nevertheless the reported first-pass success rates with direct laryngoscopy by Breeman et al. in medical CPR cases without a HEMS crew on scene shows more or less the same first-pass successes rates as in this study. The first-pass successes rates especially in patients primarily intubated by HEMS nurses or HEMS physicians may be lower due to selection bias of more complex cases in which EMS asked for P-HEMS assistance.

Moreover, we had no information on the previous experience of the individual ambulance nurse, HEMS nurse and HEMS physician and included them as groups although their experience and exposure may vary. In the group of HEMS physicians there were no differences in first-pass and overall success rates, but in previous literature on our team, trauma surgeons tended to be more reluctant to perform ETI than anaesthesiologists in similar cases.^22^ Furthermore, if intubation is indicated, trauma surgeons tend to perform the ETI themselves instead of supervising an ambulance nurse or HEMS nurse in the procedure. This may lead to a different patient selection.

Missing data in the database also confer a risk of bias although the excluded cases did not differ from the included cases in patient characteristics. In addition, these findings are consistent with a previous retrospective report by Peters et al., concerning first-pass success in Dutch prehospital care and with international results on unrecognised oesophageal intubations [5, 9].

In our HEMS operation, the choice for direct or video laryngoscopy was at the discretion of the provider, which could be a source of bias as well. Even more because we had no data on whether video laryngoscopy was used as a rescue strategy when direct laryngoscopy failed or vice versa. Ambulance nurses didn’t have a choice between these devices and had to work with the equipment supplied by their company if HEMS was not at the scene. However, for all providers, the first-pass success rates in the video laryngoscopy group were better than in the direct laryngoscopy group under supervision of a HEMS physician. Most patients were intubated on scene in the position in which they were found with limited adjustments in positioning if no cervical spine fractures were suspected. The standardised positioning on a trolley and protocolized intubation procedures as used in the UK is not a standard operating procedure in the Netherlands.

## Conclusion

First-pass and overall success rates for ETI by ambulance nurses and HEMS nurses are higher with video laryngoscopy than direct laryngoscopy. The incidence of unrecognised oesophageal intubation in cases intubated before HEMS arrived was 17.5%. The incidence in cases of circulatory arrest was higher than in circulating patients. The incidence in cases of medical cardiac arrest intubated with direct laryngoscopy was 20%. None were reported in EMS regions using video laryngoscopy for ETI during medical cardiac arrests. In traumatic circulatory arrest the incidence was higher with video laryngosopy than with direct laryngoscopy. Despite the higher first-pass and overall success rates with videolaryngoscopy, the success rates are too low to justify the current approach of airway management with ETI by ambulance nurses in the Netherlands. If training cannot be increased to improve success rates with direct or video laryngoscopy, LMA’s should be preferred by Dutch ambulance nurses as the first choice in prehospital airway manamgent.
